# β-Carotene Inhibits Expression of Matrix Metalloproteinase-10 and Invasion in *Helicobacter pylori*-Infected Gastric Epithelial Cells

**DOI:** 10.3390/molecules26061567

**Published:** 2021-03-12

**Authors:** Suji Bae, Joo Weon Lim, Hyeyoung Kim

**Affiliations:** Department of Food and Nutrition, BK21 FOUR, College of Human Ecology, Yonsei University, Seoul 03722, Korea; sestnwlrk@naver.com (S.B.); jwlim11@yonsei.ac.kr (J.W.L.)

**Keywords:** β-carotene, gastric epithelial cells, *Helicobacter pylori*, invasion, matrix metalloproteinases, peroxisome-proliferator activator receptor-γ, reactive oxygen species

## Abstract

Matrix metalloproteinases (MMPs), key molecules of cancer invasion and metastasis, degrade the extracellular matrix and cell–cell adhesion molecules. MMP-10 plays a crucial role in *Helicobacter pylori*-induced cell-invasion. The mitogen-activated protein kinase (MAPK) signaling pathway, which activates activator protein-1 (AP-1), is known to mediate MMP expression. Infection with *H. pylori*, a Gram-negative bacterium, is associated with gastric cancer development. A toxic factor induced by *H. pylori* infection is reactive oxygen species (ROS), which activate MAPK signaling in gastric epithelial cells. Peroxisome proliferator-activated receptor γ (PPAR-γ) mediates the expression of antioxidant enzymes including catalase. β-Carotene, a red-orange pigment, exerts antioxidant and anti-inflammatory properties. We aimed to investigate whether β-carotene inhibits *H. pylori*-induced MMP expression and cell invasion in gastric epithelial AGS (gastric adenocarcinoma) cells. We found that *H. pylori* induced MMP-10 expression and increased cell invasion via the activation of MAPKs and AP-1 in gastric epithelial cells. Specific inhibitors of MAPKs suppressed *H. pylori*-induced MMP-10 expression, suggesting that *H. pylori* induces MMP-10 expression through MAPKs. β-Carotene inhibited the *H. pylori*-induced activation of MAPKs and AP-1, expression of MMP-10, and cell invasion. Additionally, it promoted the expression of PPAR-γ and catalase, which reduced ROS levels in *H. pylori*-infected cells. In conclusion, β-carotene exerts an inhibitory effect on MAPK-mediated MMP-10 expression and cell invasion by increasing PPAR-γ-mediated catalase expression and reducing ROS levels in *H. pylori*-infected gastric epithelial cells.

## 1. Introduction

*Helicobacter pylori* (*H. pylori*) is a Gram-negative bacterium that infects nearly half of the world’s population. It is a human pathogen that causes stomach diseases such as gastric inflammation, ulcers, and gastric cancer [1]. *H. pylori* infection enhances the migration and invasion of gastric cells, which is closely associated with gastric cancer development [2]. Matrix metalloproteinases (MMPs) are zinc-dependent proteinases that can degrade the extracellular matrix (ECM) and cell–cell adhesion molecules [3]. They can promote cancer progression by increasing cancer-cell growth, migration, invasion, and metastasis. Among these, invasion is an important feature of malignant cancer progression [4]. *H. pylori* infection increases the expression and secretion of various MMPs, including MMP-1 [5,6], MMP-9 [7,8], MMP-7 [9], and MMP-10 [6,10], in the gastric epithelial cells or gastric cancer cells.

Among the MMPs, MMP-10 cleaves numerous ECM components, including fibronectin, proteoglycans, gelatins, and collagens [11]. Since MMPs are synthesized as inactive zymogens (proMMP) and subsequently activated by many factors to degrade the ECM, the activation of pro-MMP is linked to cancer development. MMP-10 cleaves pro-MMPs, including proMMP-1, proMMP-7, and proMMP-9 [12,13,14]. Therefore, the expression of MMP-10 has a critical role in cancer cell invasion. As signaling pathways for MMP expression, *H. pylori* infection induces MMP-1 expression via c-Jun *N*-terminal kinase (JNK) and extracellular-signal-regulated kinase (ERK) pathways in gastric cancer cells [5] and MMP-10 expression via the ERK pathway in gastric epithelial cells. [10].

*H. pylori* increases the production of reactive oxygen species (ROS) in gastric epithelial cells, which affects signal transduction in the gastric epithelia, resulting in gastric carcinogenesis [15,16,17]. ROS mediate *H. pylori*-induced activation of mitogen-activated protein kinases (MAPKs). Activated MAPKs (JNK, p38, and ERK) translocate to the nucleus, where they regulate the activity of transcription factors, including activator protein-1 (AP-1) [18,19,20]. As one of the MMP inducers, ROS upregulate MMPs by regulating both gene expression and proenzyme activation [21,22]. In cancer cells, ROS increase MMP activity by growth factor stimulation [21]. The AP-1 plays a dominant role in the transcriptional activation of MMP promoters [23]. These studies suggest the ROS-MAPKs-AP-1 MMPs axis in *H. pylori*-infected gastric epithelial cells. Therefore, reducing ROS levels in the infected tissues/cells may prevent *H. pylori*-induced MMP expression which leads to cell invasion.

Peroxisome-proliferator activator receptor gamma (PPAR-γ) is a ligand-activated nuclear receptor transcription factor that modulates inflammatory activity [24]. PPAR-γ is expressed and functionally active in gastric epithelial cells. Previously, we showed that PPAR-γ regulates the oxidative stress-induced inflammatory response by inducing the expression of antioxidant enzymes, such as catalase, in *H. pylori*-infected gastric epithelial cells [25]. Several studies have demonstrated the inhibitory effect of PPAR-γ ligands or activators on MMP-2 and MMP-9 expression in human myeloid leukemia cells [26] and human bronchial epithelial cells [27]. Taken together, decreasing ROS may be caused by the activation and expression of PPAR-γ-target gene catalase, which may reduce MMP expression in *H. pylori*-infected gastric epithelial cells.

β-Carotene is a red-orange pigment, which is abundant in fungi, plants, and fruits. It is a tetra-terpenoid consisting of a C40 structure including two β-ionone rings. It is among the most frequently consumed dietary carotenoids in human subjects [28,29] and ranking among the highest in serum concentrations [29,30]. Dietary β-carotene is cleaved at its central double bond to yield retinal by β-carotene 15,15′-dioxygenase. Retinal can be converted to retinol (the main active form of vitamin A in the blood). Retinol is oxidized to the biologically active hormone all-trans retinoic acid after delivery to peripheral cells via two steps: first, retinol dehydrogenase (which catalyzes oxidation of retinol to retinal), and second, retinal dehydrogenase (which oxidizes retinal to retinoic acid), respectively [31]. In addition to the conversion to retinal, β-carotene shows potent antioxidant activity by directly quenching singlet oxygen and lipid peroxides [32,33].

β-Carotene inhibits lung metastasis in mice by scavenging free radicals [34]. We previously demonstrated that β-carotene inhibits *H. pylori*-induced hyper-proliferation of gastric epithelial cells by suppressing β-catenin signaling and oncogene expression [35] and inducible nitric oxide synthase and cyclooxygenase-2 by reducing ROS levels [36]. Since ROS activate MAPKs and AP-1, the antioxidant effect of β-carotene may suppress *H. pylori*-induced MAPK and AP-1 signaling in AGS cells.

It has been shown that β-carotene upregulates the PPAR-γ-mediated expression of antioxidant enzymes [37,38]. Therefore, we hypothesized that β-carotene may suppress *H. pylori*-induced ROS generation and MAPK-mediated MMP-10 expression by activating PPAR-γ in gastric epithelial cells.

This study aimed to determine whether (a) β-carotene inhibits MMP-10 expression by suppressing the ROS-mediated activation of MAPKs and AP-1 and (b) whether β-carotene induces PPAR-γ-mediated catalase expression in *H. pylori*-infected AGS cells.

## 2. Results

### 2.1. H. pylori Activates MAPKs and Expression of MMP-10 in AGS Cells

**Figure 1:**
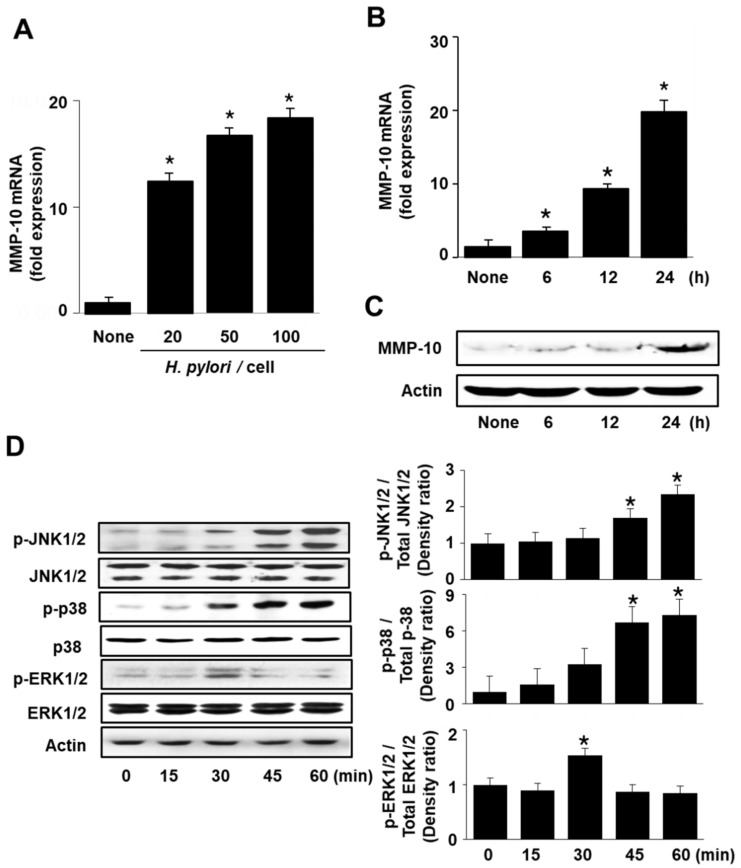
*H. pylori* induces the expression of MMP-10 and activation of MAPKs in AGS cells. (**A**) Cells were infected with *H. pylori* at the indicated ratios (*H. pylori*/cells) for 24 h. (**B**–**D**) Cells were infected with *H. pylori* at a 1:50 ratio for the indicated time periods. (**A**,**B**) The expression of MMP-10 mRNA was analyzed by real-time PCR and normalized to β-actin mRNA. All data are shown as the mean ± standard error (S.E.) of three independent experiments. * *p* < 0.05 vs. none (cells without any treatment or infection). (**C**) Protein levels of MMP-10 were determined by Western blot analysis, using actin as the loading control. (**D**) Protein levels of phosphorylated or total form of JNK1/2, p38 and ERK1/2 were determined by Western blot analysis. Actin served as a loading control (left panel). Right panel: the densitometry data represent means ± S.E. from three immunoblots and are shown as relative density of phosphorylated protein band normalized to total form of protein level. * *p* < 0.05 vs. 0 min.

First, we wanted to investigate whether *H. pylori* induces mRNA and MMP-10 protein expression by real-time polymerase chain reaction (PCR) and Western blot analysis, respectively. AGS cells were infected with *H. pylori* at the indicated ratios. At 24 h, the MMP-10 mRNA was upregulated by *H. pylori* in a density-dependent manner (Figure 1A). At a 50:1 bacteria/cell ratio, *H. pylori* increased the mRNA and protein levels of MMP-10 in a time-dependent manner. The maximum induction of MMP-10 in *H. pylori*-infected cells was observed at 24 h (Figure 1B,C). To determine whether *H. pylori* activates the MAPK signaling pathway, phosphorylated and total forms of MAPKs were detected by Western blotting. *H. pylori* increased the levels of phosphorylated MAPKs (p-JNK1/2, p-p38, and p-ERK1/2) in AGS cells at 30 min, while the total levels were not changed (Figure 1D). Levels of both p-JNK1/2 and p-38 steadily increased till 60 min but p-ERK1/2 decreased after 30 min.

### 2.2. MAPK Inhibitors Prevent H. pylori-Induced Expression of MMP-10 in AGS Cells

**Figure 2:**
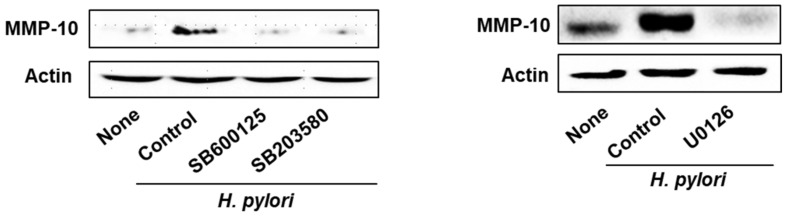
JNK, p38, and ERK inhibitors reduced *H. pylori*-induced MMP-10 expression in AGS cells. The cells were pretreated with SB600125 (a JNK inhibitor, 20 µM) or SB203580 (a p38 inhibitor, 20 µM) for 60 min, or U0126 (an ERK inhibitor, 10 µM) for 30 min, and then infected with *H. pylori* for 24 h. MMP-10 levels were determined by Western blot analysis. Actin was used as a loading control.

To confirm the involvement of MAPKs in the *H. pylori*-induced expression of MMP-10, we used MAPK inhibitors SB600125 (JNK inhibitor), SB203580 (p38 inhibitor), and U0126 (ERK inhibitor). AGS cells were pretreated with SB600125 for 60 min, SB203580 for 60 min, and U0126 for 30 min, and infected with *H. pylori* for 24 h. All three MAPK inhibitors suppressed *H. pylori*-induced MMP-10 expression in AGS cells (Figure 2). These results indicate that *H. pylori* induces MMP-10 expression through JNK, p38, and ERK signaling in AGS cells.

### 2.3. β-Carotene Inhibits H. pylori-Induced Activation of MAPKs and AP-1, and Expression of MMP-10 in AGS Cells

**Figure 3:**
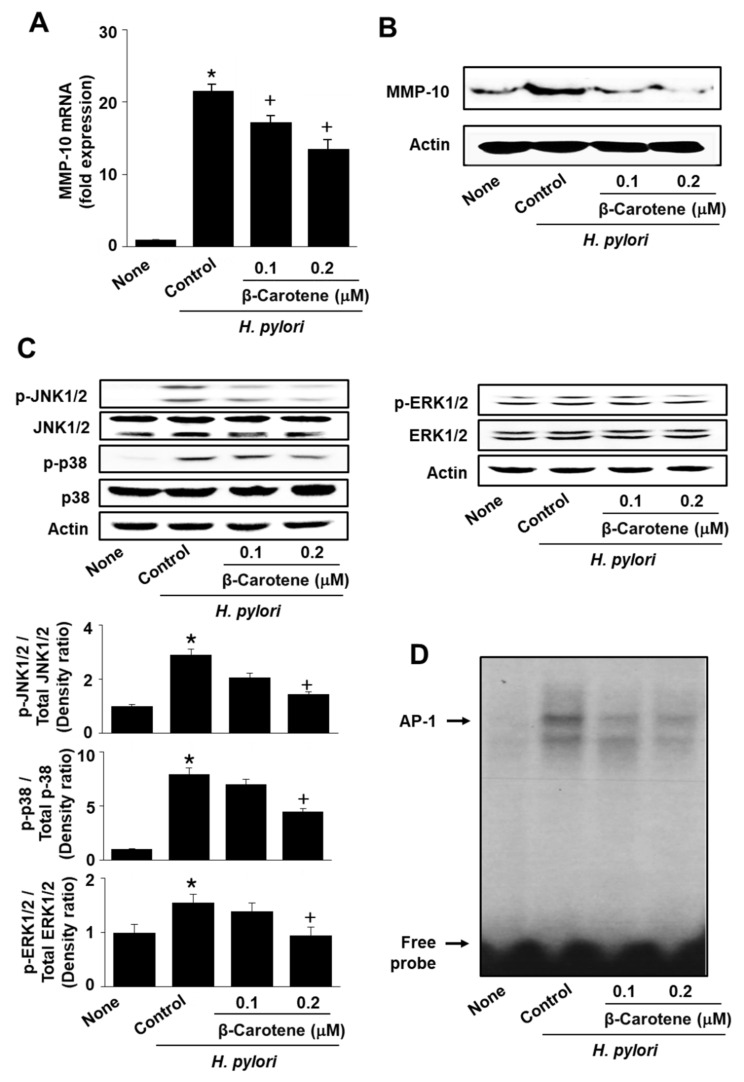
β-Carotene inhibits *H. pylori*-induced activation of MAPKs and AP-1, and expression of MMP-10 in AGS cells. Cells were pretreated with the indicated concentrations of β-carotene for 2 h and then infected with *H. pylori* for 24 h (**A**,**B**), 1 h (**C**, left panel), 30 min (**C**, right panel), and 1 h (**D**). (**A**) MMP-10 mRNA expression was analyzed by real-time PCR and normalized to β-actin mRNA. All data are shown as the mean ± S.E. of three independent experiments. * *p* < 0.05 vs. none (cells without any treatment or infection); + *p* < 0.05 vs. control (cells with *H. pylori* infection alone). (**B**) The level of MMP-10 was determined by Western blot analysis, using actin as the loading control. (**C**) Protein levels of phosphorylated or total form of JNK1/2, p38, and ERK1/2 were determined by Western blot analysis. Actin served as a loading control (upper panel). Lower panel: The densitometry data represent means ± S.E. from three immunoblots and are shown as relative density of phosphorylated protein band normalized to total form of protein level. * *p* < 0.05 vs. none (cells without any treatment or infection); + *p* < 0.05 vs. control (cells with *H. pylori* infection alone). (**D**) The DNA binding activity of AP-1 was measured by an electrophoretic mobility shift assay (EMSA).

Next, we examined the effect of β-carotene on the *H. pylori*-induced expression of MMP-10. AGS cells were infected with *H. pylori* in the presence or absence of β-carotene. β-Carotene inhibited *H. pylori*-induced mRNA and the protein expression of MMP-10 at 24 h (Figure 3A,B). This prompted us to examine whether the inhibitory effect of β-carotene is mediated by suppressing MAPK activation. Levels of phosphorylated and total forms of MAPKs were determined in cells treated with or without β-carotene and infected with *H. pylori*. As shown in Figure 3C, β-carotene markedly reduced the phosphorylation of JNK1/2, p38, and ERK1/2 in a dose-dependent manner. Since MAPK induction is an upstream signaling event in AP-1-mediated MMP-10 expression, AP-1 DNA-binding activity was determined in the nuclear extracts of cells treated with or without β-carotene and infected with *H. pylori* (Figure 3D). β-Carotene inhibited *H. pylori*-induced AP-1 activation in AGS cells These results suggest that β-carotene inhibits the activation of MAPKs and AP-1 and thus, MMP-10 expression in *H. pylori*-infected AGS cells.

### 2.4. β-Carotene Inhibits Cell Invasion Induced by H. pylori in AGS Cells

**Figure 4:**
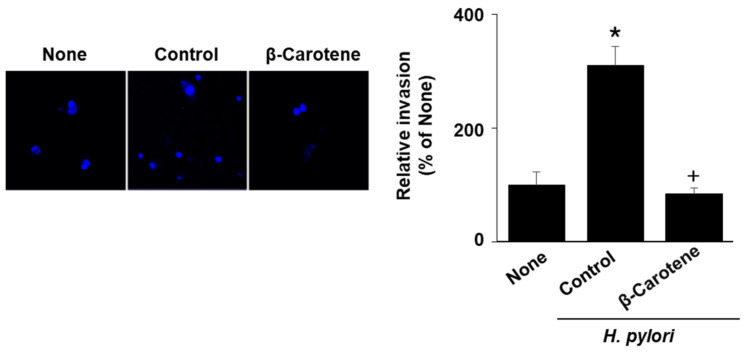
β-Carotene inhibits cell invasion induced by *H. pylori* in AGS cells. AGS cells were pretreated with β-carotene (0.2 µM) for 2 h and then infected with *H. pylori* for 24 h. Invasive cells were measured by staining them on Matrigel-coated filters with 4′,6-diamidino-2-phenylindole (DAPI) and visualizing them under a confocal laser scanning microscope (left panel). The graph represents the relative percentage of invasive cells (right panel). All data are shown as the mean ± S.E. of three independent experiments. The percentage of invasive cells in none (the cells without any treatment or infection) was set as 100%. * *p* < 0.05 vs. none (cells without any treatment or infection); + *p* < 0.05 vs. control (cells with *H. pylori* infection alone).

To determine whether β-carotene inhibits cell invasion in *H. pylori*-infected cells, AGS cells were infected with *H. pylori* in the presence or absence of β-carotene and analyzed using a cell-invasion assay. *H. pylori* significantly increased cell invasion, which was inhibited by β-carotene (Figure 4). These results demonstrate that β-carotene inhibits cell invasion induced by *H. pylori* infection.

### 2.5. β-Carotene Upregulates PPAR-γ and Catalase and Reduces ROS Levels in H. pylori-Infected AGS Cells

**Figure 5:**
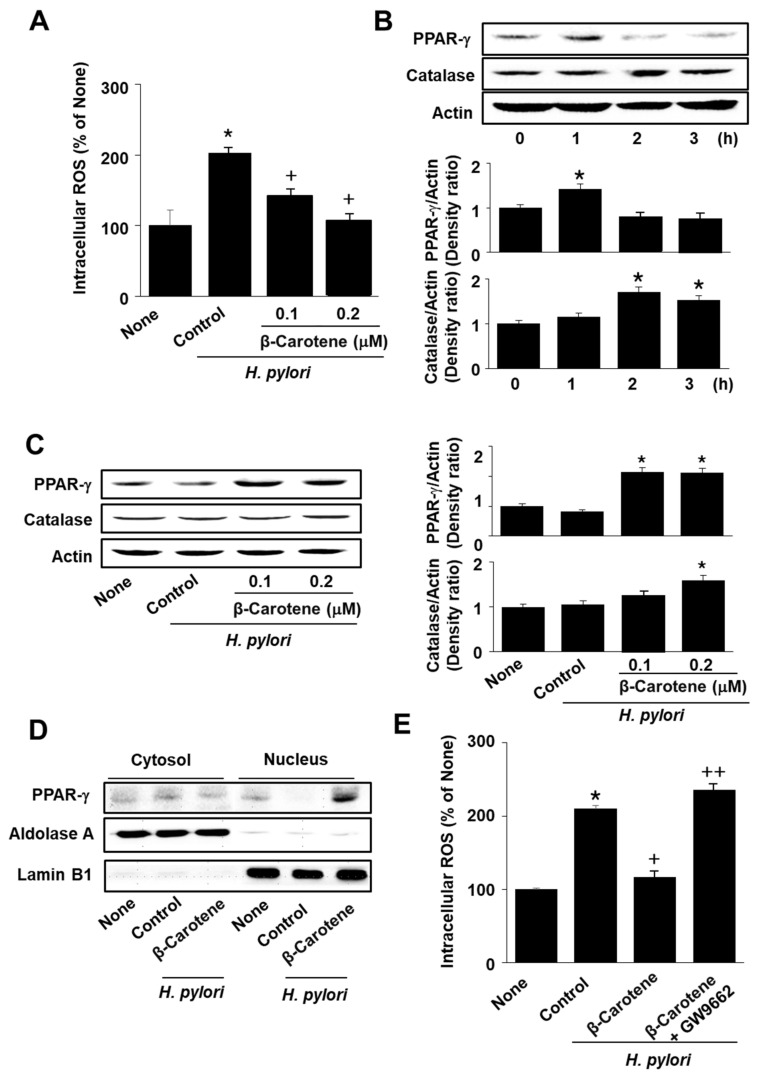
β-Carotene reduces intracellular ROS levels and induces expression of PPAR-γ and catalase in *H. pylori*-infected AGS cells. (**A**,**C**) Cells were pretreated with the indicated concentrations of β-carotene for 2 h and then infected with *H. pylori* for 30 min. (**B**) Cells were treated with β-carotene (0.2 µM) for the indicated time period without *H. pylori* infection. (**D**) Cells were pretreated with β-carotene (0.2 µM) for 2 h and then infected with *H. pylori* for 30 min. (**E**) Cells were co-treated with the PPAR-γ antagonist GW9662 (5 μM) and β-carotene (0.2 µM) for 2 h and then infected with *H. pylori* for 30 min. (**A**,**E**) Intracellular ROS levels were measured by dichlorofluorescein (DCF) fluorescence. All data are shown as the mean ± S.E. of three independent experiments. ROS levels in none (the cells without any treatment or infection) were set as 100%. * *p* < 0.05 vs. none (cells without any treatment or infection); + *p* < 0.05 vs. control (cells with *H. pylori* infection alone); ++ *p* < 0.05 vs. cells with β-carotene treatment and *H. pylori* infection. (**B**,**C**) Protein levels of PPAR-γ and catalase in whole-cell extracts were determined by Western blot analysis, using actin as the loading control. The densitometry data represent means ± S.E. from three immunoblots and are shown as relative density of protein band normalized to actin level. * *p* < 0.05 vs. 0 h (**B**) or control (cells with *H. pylori* infection alone) (**C**). (**D**) Levels of PPAR-γ were measured by Western blot analysis. Aldolase was used as a marker of cytosol, while lamin B was used as a nuclear marker.

Finally, we determined the antioxidant effect and the mechanisms underlying β-carotene in *H. pylori*-infected cells. Intracellular ROS levels and the expression of PPAR-γ and catalase were measured in cells treated with or without β-carotene (0.2 µM), and infected with *H. pylori*. As shown in Figure 5A, β-carotene reduced ROS levels, which were increased by *H. pylori* infection in AGS cells. Treatment with β-carotene (0.2 µM) increased the expression of PPAR-γ at 1 h, but increased the expression of catalase, a PPAR-γ target gene, till 3 h, in the absence of *H. pylori* (Figure 5B). *H. pylori* infection reduced the expression of PPAR-γ, which was prevented by β-carotene (Figure 5C). Additionally, *H. pylori* infection reduced the nuclear levels of PPAR-γ, which was restored by β-carotene treatment (Figure 5D).

To scrutinize the involvement of PPAR-γ in the antioxidant mechanisms of β-carotene, *H. pylori*-infected cells were treated with the PPAR-γ antagonist GW9662 and β-carotene. Co-treatment with GW9662 reversed the effect of β-carotene on *H. pylori*-induced intracellular ROS production (Figure 5E). These results indicate that β-carotene reduces ROS levels by activating PPAR-γ and inducing the expression of catalase in *H. pylori*-infected cells.

## 3. Discussion

Gastric cancer is the fourth leading cause of cancer. Infection with *H. pylori* is a significant risk factor for chronic gastritis, peptic ulcers, and gastric carcinogenesis. MMP expression is involved in the invasion and metastasis of malignant tumors, and is induced in *H. pylori*-infected gastric epithelial cells [5,6,7,8,9,10]. High levels of plasma MMPs were found in gastric cancer patients and, therefore, MMPs could serve as important diagnostic markers of gastric cancer [39]. MMP-10 is upregulated via ERK signaling in *H. pylori*-infected AGS cells [10]. Consistent with this study, we observed higher MMP-10 expression in AGS cells following *H. pylori* infection. In addition to ERK, JNK and p38 are activated, which induces MMP-10 expression in *H. pylori*-infected cells.

Cytokines, growth factors, and phorbol esters activate a group of protein kinases (MAPK kinase kinases (MAPKKKs)) that phosphorylate other kinases (MAPK kinases (MAPKKs)), which in turn are responsible for the phosphorylation and activation of MAPK. Upon activation by MAPKKs, MAPKs translocate to the nucleus to phosphorylate and activate various transcription factors [40,41]. Of particular relevance to MMP transcription, JNKs and ERKs phosphorylate and activate AP-1 family member c-Jun [42,43], which dimerizes with c-Fos to drive the transcription of multiple MMP genes. In the present study, we found that the *H. pylori*-induced activation of MAPKs mediates AP-1 activation and MMP-10 expression in AGS cells.

ROS have been implicated in the development of gastric cancer. The overproduction of ROS, which leads to the activation of pro-survival signaling pathways, including cancer cell proliferation and invasion, is induced by *H. pylori* infection [16]. Higher ROS levels activate MAPK signal transduction and the transcription factor, AP-1, in *H. pylori*-infected cells [18,19]. AP-1 transcription factor is important in regulating MMP family members and is sensitive to regulation by redox conditions [21]. Even though ROS generally increase the AP-1 level for the activation of MMP gene expression, ROS can also reduce the transcriptional activity of AP-1-through direct oxidation of cysteine residues contained within the DNA-binding domain [44]. Therefore, the cycling of cysteinyl residues is among the oxidant-dependent mechanisms that regulate the activity of AP-1. In fact, AP-1 DNA-binding activity increases after the reducing agent thioredoxin treatment in monocytes. When the conserved cysteine in the basic binding motif of both Fos and Jun is oxidized, the binding of these proteins to AP-1 sequences is diminished. On the other hand, if Fos/Jun heterodimers are bound to an AP-1 site, they cannot be oxidized [45]. In the present study, *H. pylori* infection increased both ROS levels and AP-1-DNA binding activity in gastric epithelial cells. Therefore, cysteine residues in the DNA-binding domain may not be affected nor oxidized by *H. pylori* infection at the infection ratio of 1:50 (cells: bacterium). On the other hand, Fos/Jun heterodimers may be bound directly to the AP-1 site by MAPK activation in *H. pylori*-infected cells. Further study should be performed to investigate whether a high infection ratio (more than 1:50, cells: bacterium) causes the oxidation of critical cysteine residues in the DNA-binding domain for AP-1 in gastric epithelial cells.

β-Carotene is the most frequently found carotenoid in the human diet. In the human body, β-carotene is absorbed, distributed, and metabolized by enzymatic and/or non-enzymatic oxidant cleavage into several metabolites. Despite the broadly accepted biological value of β-carotene, it is a double-edged sword, due to its potential antioxidant versus pro-oxidant behavior. Regarding the pharmacological role of β-carotene, a high concentration (50 and 100 µM) of β-carotene increases ROS levels and caspase-3 activity, which results in a reduction in DNA rapier protein Ku protein and apoptosis in gastric cancer AGS cells [46]. Furthermore, 100 µM of β-carotene induces apoptosis by increasing apoptotic protein p53 and decreasing anti-apoptotic Bcl-2 as well as nuclear ataxia-telangiectasia mutated (ATM), a sensor for DNA-damaging agents, in AGS cells [47]. Cui et al. [37] suggested that the chemopreventive activity of a high concentration of β-carotene (>20 µM) is associated with ROS production, which results in mitochondrial dysfunction, cytochrome c release, and apoptosis in human breast cancer MCF-7 cells. Palozza et al. [48] demonstrated that β-carotene (>10 µM) induced ROS production and apoptosis in human leukemic HL-60 cells. In addition, β-carotene (50–100 µM) increases ROS levels and inhibits cell growth with the expression of c-myc in colon adenocarcinoma WiDr cells. Thus, a high concentration of β-carotene may exert pro-oxidant activity, which contributes to anti-cancer effects.

However, our previous study showed that a low concentration (0.5 and 1 µM) of β-carotene reduced ROS levels and inhibited NF-B activation, NF-B-regulated expression of tumor necrosis factor receptor associated factor (TRAF), and hyper-proliferation in *H. pylori*-infected gastric epithelial cells [49]. A low concentration of β-carotene (0.15 and 0.3 µM) inhibited peroxynitrite anion-induced lipid peroxide production in rat brain synaptosomes [50]. Therefore, we used 0.1 and 0.2 µM of β-carotene to determine the antioxidant mechanism of β-carotene in relation to MMP expression in *H. pylori*-infected gastric epithelial cells in the present study.

In the present study, we found that β-carotene reduced ROS levels, which was increased by *H. pylori* infection. β-Carotene reduced cell invasion as well as MMP-10 expression in *H. pylori*-infected cells. The *H. pylori*-induced activation of MAPKs and AP-1 was inhibited by β-carotene. The JNK, p38, and ERK inhibitors suppressed *H. pylori*-induced MMP-10 expression. These results indicate that β-carotene inhibits *H. pylori*-induced invasion by downregulating the MAPK-mediated activation of AP-1, which in turn reduces MMP-10 expression in gastric epithelial cells.

Regarding the studies on β-carotene and MMP expression and invasion, β-carotene attenuates invasion and metastasis in neuroblastoma cells in vitro and in vivo [51]. Dietary β-carotene inhibits peroxidized cholesterol-induced MMP-9 activation in hairless mouse skin [52]. In keratinocytes, β-carotene dose-dependently quenches the singlet oxygen-mediated induction of MMP-1 and MMP-10 [53]. β-Carotene treatment downregulates the expression of MMP-2 and MMP-9 by suppressing the nuclear translocation of p65, p50, c-Rel subunits of nuclear factor-kappa B, and other transcription factors, such as c-fos, a component of AP-1, in B16F-10 melanoma cells [54]. β-Carotene reduces invasiveness with a decreased expression of MMP-7 and MMP-28 in colorectal carcinoma cells [55].

For the antioxidant mechanism of β-carotene, we found that β-carotene induces the expression and nuclear translocation of PPAR-γ, which promotes catalase expression in *H. pylori*-infected AGS cells. These data demonstrate that β-carotene reduces ROS levels through the upregulation of the PPAR-γ–mediated expression of the antioxidant enzyme, catalase. Cui et al. [37] demonstrated that β-carotene upregulates PPAR-γ expression, but increases ROS production in MCF-7 breast cancer cells. Kaliappan et al. [56] demonstrated that dietary β carotene inhibits Angiotensin II-induced renal damage by repressing PPARγ and renin 1 in apo E knockout mice. Therefore, the effect of β-carotene on PPAR-γ expression and activation may be different depending on the tissues, stimuli, and environmental and genetic factors. Further studies should be performed to determine the interaction of PPAR-γ, ROS, and antioxidant enzyme expression on *H. pylori*-associated gastric cancer development.

Recently, Mao et al. [57] screened epidemiological studies and demonstrated that increased intake of phytochemicals in fruits and vegetables could reduce the risk of gastric cancer. They suggested that phytochemicals have the potential for the prevention and management of gastric cancer in humans. This study supports the present finding showing that dietary β-carotene, which is abundant in fruits and vegetables, may reduce *H. pylori*-associated gastric cancer incidence by reducing the oxidative stress-mediated MMP-10 expression and invasive property of gastric epithelial cells.

In conclusion, β-carotene activates PPAR-γ and induces its downstream target, catalase, which leads to a reduction in ROS levels and the inhibition of JNK/p38/ERK signaling in *H. pylori*-infected gastric epithelial cells. Through this mechanism, β-carotene significantly suppressed *H. pylori*-induced MMP-10 expression and reduced the invasive phenotype of infected gastric epithelial cells.

## 4. Materials and Methods

### 4.1. Cell Line and Culture Conditions

Human gastric epithelial AGS cells were purchased from the American Type Culture Collection (Rockville, MD, USA). The cells were cultured in complete medium consisting of Roswell Park Memorial Institute (RPMI) 1640 medium (GIBCO, Grand Island, NY, USA) with 10% fetal bovine serum (FBS), 2 mM glutamine, 100 units/mL penicillin, and 100 µg/mL streptomycin (Sigma-Aldrich, St. Louis, MO, USA). The cells were cultured at 37 °C and maintained in a humidified atmosphere containing 5% CO_2_.

### 4.2. Bacterial Strain and Growth Conditions

All experiments were performed with *H. pylori*, cag A positive strain 60190 (ATCC 49503). Chocolate agar plates (Becton Dickinson Microbiology Systems, Cockeysvile, MD, USA) were used to grow the bacteria at 37 °C under microaerophilic conditions using an anaerobic chamber (BBL Campy Pouch^®^ System, Becton Dickinson Microbiology Systems, Franklin Lakes, NJ, USA).

### 4.3. Reagents

β-Carotene (Sigma-Aldrich, St. Louis, MO, USA) was dissolved in THF (final concentration 10 mM) and diluted in media to the desired concentrations. The PPAR-γ antagonist GW9662 (Sigma-Aldrich) was dissolved in DMSO. The MAPK inhibitors, U0126 (an ERK inhibitor; Catalog #9903, Cell Signaling Technology, Inc., Beverly, MA, USA), SB203580 (a p38 inhibitor; Catalog #559389, Calbiochem Biochemicals, San Diego, CA, USA), and SB600125 (a JNK inhibitor; Catalog #10010466, Cayman Chemical, Ann Arbor, MI, USA), were dissolved in DMSO.

### 4.4. Infection of AGS Cells with H. pylori

AGS cells were seeded and cultivated overnight to reach 80% confluency. *H. pylori* were harvested from the chocolate agar plates and resuspended in antibiotic-free RPMI 1640 medium supplemented with 10% FBS. Subsequently, AGS cells were infected with *H. pylori* at multiplicity-of-infection of 50:1.

### 4.5. Experimental Protocols

To investigate the effect of β-carotene, AGS cells (1.5 × 10^5^/2 mL, 7.0 × 10^5^/10 mL) were pretreated with β-carotene (0.1 or 0.2 µM) for 2 h and then infected with *H. pylori* for 30 min (for ROS levels, PPAR-γ DNA binding activity, protein levels of PPAR-γ and catalase), 1 h (for protein expression of JNK/p38/ERK and DNA binding activity of AP-1), and 24 h (for mRNA expression and protein levels of MMP-10, and invasion assay). To determine the involvement of MAPKs, the MAPK inhibitors SB203580 and SP600125 were added to the culture medium 1 h before infection with *H. pylori*. In the case of U0126, the cells were pretreated with U0126 for 30 min before infection with *H. pylori*.

### 4.6. Preparation of Cell Extracts

The cells were collected by treatment with trypsin-ethylenediaminetetraacetic acid (EDTA), followed by centrifugation at 1000× *g* for 5 min. The cell pellets were resuspended in lysis buffer, including 10 mM Tris pH 7.4, 15 mM NaCl, 1% NP-40, and protease inhibitor cocktail (Complete; Roche, Mannheim, Germany), and lysed by passing the cells through a 1 mL syringe several times. The resulting mixture was incubated on ice for 30 min and centrifuged at 13,000× *g* for 15 min. The supernatants were collected and used as whole-cell extracts. The cytosolic and nuclear extracts were prepared using a NE-PER^®^ nuclear and cytoplasmic extraction kit (Thermo Fisher, Waltham, MA, USA) according to the manufacturer’s instructions. In brief, cells were resuspended in the cytoplasmic extraction reagent containing protease inhibitors and vortexed for 15 s, followed by centrifugation at 13,000× *g* for 10 min. The supernatants were used as cytosolic extracts. The nuclear pellet was resuspended in nuclear extraction reagent on ice, and then centrifuged at 13,000× *g* for 10 min. The supernatants were collected and used as nuclear extracts. The specificity of the nuclear extracts was confirmed by the predominant presence of lamin B1 in the nuclear fraction. A Bradford assay (Bio-Rad Laboratories, Hercules, CA, USA) was used to determine the protein concentrations.

### 4.7. Real-Time PCR Analysis

Total RNA was isolated using TRI reagent (Molecular Research Center, Inc., Cincinnati, OH, USA). The isolated RNA was reverse transcribed into cDNA using random hexamers and MuLV reverse transcriptase (Promega, Madison, WI, USA) at 23 °C for 10 min, 37 °C for 60 min, and 95 °C for 5 min. For real-time PCR, the cDNA was amplified with specific primers for human MMP-10 and β-actin. For the MMP-10 PCR product, 5′-CATTCCTTGTGCTGTTGTGTC-3′ (forward primer) and 5′-TGTCTAGCTTCCCGTTCACC-3′ (reverse primer) were used. The sequence of β-actin primers was the following: 5′-ACCAACTGGGACGACATGGAG-3′ (forward primer) and 5′-GTGAGGATCTTCATGAGGTAGTC-3 (reverse primer). The cDNA was amplified by 45 cycles including denaturation at 95 °C for 30 s, annealing at 55 °C for 30 s, and extension at 72 °C for 30 s. During the first cycle, the 95 °C step was extended to 3 min. Amplification of the β-actin gene was performed in the same reaction and served as the reference gene.

### 4.8. Invasion Assay

Invasion of AGS cells infected with *H. pylori* was evaluated in Matrigel-coated 24-well invasion chambers (#354480, Corning Life Sciences, Teterboro, NJ, USA). The upper and lower compartments were separated by Matrigel-coated filters with 8 μm pore size. AGS cells (4.0 × 10^4^ cells/well) were seeded on the reconstituted basement membrane in RPMI 1640 supplemented with 0.01% FBS. Following infection with *H. pylori*, the cells were incubated for 24 h in the upper chamber with or without β-carotene. Cells which crossed the filter and attached to the lower surface of the Matrigel-coated membrane (invasive cells) were washed with PBS and fixed in 4% formaldehyde for 30 min at room temperature. The cells were rinsed twice with PBS and then stained using 4′,6-diamidino-2-phenylindole (DAPI) for 30 min. The number of cells that had migrated to the lower surface was counted in twelve random fields using a laser scanning confocal microscope (Zeiss LSM 880, Carl Zeiss Inc., Thornwood, NY, USA) (10×).

### 4.9. Measurement of Intracellular ROS Levels

For the measurement of intracellular ROS, cells were treated with 10 µg/mL of dichlorofluorescein diacetate (DCF-DA; Sigma-Aldrich, St. Louis, MO, USA) and incubated at 37 °C for 30 min. DCF fluorescence (excitation 495 nm and emission 535 nm) was measured using a Victor5 multi-label counter (PerkinElmer Life and Analytical Sciences, Boston, MA, USA). ROS levels were quantified based on the relative increase in fluorescence.

### 4.10. Electrophoretic Mobility Shift Assay (EMSA)

The PPAR-γ gel-shift oligonucleotide (5′-CAAAACTAGGTCAAAGCTCA-3′; sc-2587, Santa Cruz Biotechnology, Dallas, TX, USA) and AP-1 gel-shift oligonucleotide (5′-CGCTTGATAGTCAGCCGGAA-3′; Promega) were radiolabeled using [^32^P]-dATP (Amersham Biosciences, Piscataway, NJ, USA) and T4 polynucleotide kinase (GIBCO, Grand Island, NY, USA). The radiolabeled oligonucleotides were separated from free [^32^P]-dATP using Bio-Rad purification columns (Bio-Rad Laboratories, Hercules, CA, USA). Nuclear extracts were incubated at room temperature for 30 min under the following conditions: [^32^P]-labeled oligonucleotide in buffer containing 12% glycerol, 12 mM HEPES (pH 7.9), 1 mM EDTA, 1 mM DTT, 25 mM KCl, 5 mM MgCl_2_, and 0.04 µg/mL poly[d(I-C)]. The samples were electrophoretically separated in a nondenaturing 5% acrylamide gel. The gel was dried at 80 °C for 2 h and exposed to a radiography film at −80 °C with intensifying screens.

### 4.11. Western Blot Analysis

Whole-cell extracts (10–40 μg) were separated using 7–13% SDS polyacrylamide gel electrophoresis under reducing conditions and transferred to nitrocellulose membranes (Amersham, Inc., Arlington Heights, IL, USA) by electroblotting. The transfer of protein was verified using reversible staining with Ponceau S. Membranes were blocked with 3% non-fat dry milk in Tris-buffered saline and 0.2% Tween 20 (Santa Cruz Biotechnology, Dallas, TX, USA) (TBS-T) for 1 h at 15–25 °C. Antibodies against MMP-10 (sc-80197, Santa Cruz Biotechnology), p-ERK (sc-7383, Santa Cruz Biotechnology), ERK (#9102, Cell Signaling Technology, Danvers, MA, USA), p-JNK(#9251S, Cell Signaling Technology), JNK (#9252, Cell Signaling Technology), p-p38 (#9211S, Cell Signaling Technology), p38 (#9212, Cell Signaling Technology), PPAR-γ (sc-7273, Santa Cruz Biotechnology), catalase (ab16731, Abcam, Cambridge, UK), lamin B1 (ab16048, Abcam, Cambridge, UK), aldolase A (sc-390733, Santa Cruz Biotechnology), and actin (sc-47778, Santa Cruz Biotechnology) were diluted in TBS-T containing 3% non-fat dry milk and incubated overnight at 4 °C. Membranes were washed with TBS-T, followed by detection with horseradish peroxidase-conjugated secondary antibodies (anti-mouse or anti-rabbit). Proteins were visualized using an enhanced chemiluminescence detection system (Santa Cruz Biotechnology) through exposure to BioMax MR films (Kodak, Rochester, NY, USA).

Protein level was compared to that of the loading control actin or total form of MAPK (ERK1/2, JNK1/2, p-38). Intensity of each protein band was densitometrically quantified by using the software Image J (National Institutes of Health, USA). The densitometry data represent means ± S.E. from three immunoblots and are shown as relative density of protein band normalized to actin or total form of MAPK.

### 4.12. Statistical Analysis

One-way analysis of variance followed by the Newman–Keuls post-hoc test was used for statistical analysis. All data are reported as the mean ± S.E. of three independent experiments. For each experiment, the number of each group was 4 (*n* = 4 per each group). A *p*-value less than 0.05 was considered statistically significant.

## Data Availability

The data used to support the findings of this study are included within the article.

## Abbreviations

AP-1	Activator protein-1
Cag A	Cytotoxin-associated gene A
DCF-DA	Dichlorofluorescein diacetate
ECM	Extracellular matrix
EMSA	Electrophoretic mobility shift assay
JNK	c-Jun N-terminal kinase
ERK	Extracellular signal-regulated kinase
MAPK	Mitogen-activated protein kinase
MAPKKs	MAPK kinases
MAPKKKs	MAPK kinase kinases
MMP	Matrix metalloproteinase
PARγ	Peroxisome proliferator-activated receptor-γ
ROS	Reactive oxygen species

## References

[1] Hooi J.K.Y., Lai W.Y., Ng W.K., Suen M.M.Y., Underwood F.E., Tanyingoh D., Malfertheiner P., Graham D.Y., Wong V.W.S., Wu J.C.Y. (2017). Global prevalence of *Helicobacter pylori* infection: Systematic review and meta-analysis. Gastroenterology.

[2] Song Y., Liu G., Liu S., Chen R., Wang N., Liu Z., Zhang X., Xiao Z., Liu L. (2019). *Helicobacter pylori* upregulates TRPC6 via Wnt/β-catenin signaling to promote gastric cancer migration and invasion. OncoTargets Ther..

[3] Kessenbrock K., Plaks V., Werb Z. (2010). Matrix metalloproteinases: Regulators of the tumor microenvironment. Cell.

[4] Page-McCaw A., Ewald A.J., Werb Z. (2007). Matrix metalloproteinases and the regulation of tissue remodelling. Nat. Rev. Mol. Cell Biol..

[5] Krueger S., Hundertmark T., Kalinski T., Ulrich Peitz U., Wex T., Malfertheiner P., Naumann M., Roessner A. (2006). *Helicobacter pylori* encoding the pathogenicity island activates matrix metalloproteinase 1 in gastric epithelial cells via JNK and ERK. J. Biol. Chem..

[6] Jiang H., Zhou Y., Liao Q., Ouyang H. (2014). *Helicobacter pylori* infection promotes the invasion and metastasis of gastric cancer through increasing the expression of matrix metalloproteinase-1 and matrix metalloproteinase-10. Exp. Ther. Med..

[7] Mori N., Sato H., Hayashibara T., Senba M., Geleziunas R., Wada A., Hirayama T., Yamamoto N. (2003). *Helicobacter pylori* induces matrix metalloproteinase-9 through activation of nuclear factor κB. Gastroenterology.

[8] Nam Y.H., Ryu E., Lee D., Shim H.J., Lee Y.C., Lee S.T. (2011). CagA phosphorylation-dependent MMP-9 expression in gastric epithelial cells. Helicobacter.

[9] Wroblewski L.E., Noble P.J., Pagliocca A., Pritchard D.M., Hart C.A., Campbell F., Dodson A.R., Dockray G.J., Varro A. (2003). Stimulation of MMP-7 (matrilysin) by *Helicobacter pylori* in human gastric epithelial cells: Role in epithelial cell migration. J. Cell Sci..

[10] Costa A.M., Ferreira R.M., Pinto-Ribeiro I., Sougleri J.S., Oliveira M.J., Carreto L., Santos M.A., Sgouras D.N., Carneiro F., Leite M. (2016). *Helicobacter pylori* activates matrix metalloproteinase 10 in gastric epithelial cells via EGFR and ERK-mediated pathways. J. Infect. Dis..

[11] Frederick L.A., Matthews J.A., Jamieson L., Justilien V., Thompson E.A., Radisky D.C., Fields A.P. (2008). Matrix metalloproteinase-10 is a critical effector of protein kinase Cι-Par6α-mediated lung cancer. Oncogene.

[12] Nakamura H., Fujii Y., Ohuchi E., Yamamoto E., Okada Y. (1998). Activation of the precursor of human stromelysin 2 and its interactions with other matrix metalloproteinases. Eur. J. Biochem..

[13] Scheau C., Badarau I.A., Costache R., Caruntu C., Mihai G.L., Didilescu A.C., Constantin C., Neagu M. (2019). The role of matrix metalloproteinases in the epithelial-mesenchymal transition of hepatocellular carcinoma. Anal. Cell. Pathol..

[14] Gobin E., Bagwell K., Wagner J., Mysona D., Sandirasegarane S., Smith N., Bai S., Sharma A., Schleifer R., She J.-X. (2019). A pan-cancer perspective of matrix metalloproteases (MMP) gene expression profile and their diagnostic/prognostic potential. BMC Cancer.

[15] Kim H. (2005). Oxidative stress in *Helicobacter pylori*-induced gastric cell injury. Inflammopharmacology.

[16] Handa O., Naito Y., Yoshikawa T. (2010). *Helicobacter pylori*: A ROS-inducing bacterial species in the stomach. Inflamm. Res..

[17] Baek H.Y., Lim J.W., Kim H., Kim J.M., Kim J.S., Jung H.C., Kim K.H. (2004). Oxidative-stress-related proteome changes in *Helicobacter pylori*-infected human gastric mucosa. Biochem. J..

[18] Seo J.H., Lim J.W., Kim H., Kim K.H. (2004). *Helicobacter pylori* in a Korean isolate activates mitogen-activated protein kinases, AP-1, and NF-κB and induces chemokine expression in gastric epithelial AGS cells. Lab. Investig..

[19] Allison C.C., Kufer T.A., Kremmer E., Kaparakis M., Ferrero R.L. (2009). *Helicobacter pylori* induces MAPK phosphorylation and AP-1 activation via a NOD1-dependent mechanism. J. Immunol..

[20] Choi J.H., Cho S.O., Kim H. (2016). α-Lipoic acid inhibits expression of IL-8 by suppressing activation of MAPK, Jak/Stat, and NF-κB in *H. pylori*-infected gastric epithelial AGS cells. Yonsei Med. J..

[21] Nelson K.K., Melendez J.A. (2004). Mitochondrial redox control of matrix metalloproteinases. Free Radic. Biol. Med..

[22] Mori K., Shibanuma M., Nose K. (2004). Invasive potential induced under long-term oxidative stress in mammary epithelial cells. Cancer Res..

[23] Benbow U., Brinckerhoff C.E. (1997). The AP-1 site and MMP gene regulation: What is all the fuss about?. Matrix Biol..

[24] Polvani S., Tarocchi M., Galli A. (2012). PPARγ and oxidative stress: Con(β) catenating NRF2 and FOXO. PPAR Res..

[25] Kim S.H., Lim J.W., Kim H. (2018). Astaxanthin inhibits mitochondrial dysfunction and interleukin-8 expression in *Helicobacter pylori*-infected gastric epithelial cells. Nutrients.

[26] Liu J., Lu H., Huang R., Lin D., Wu X., Lin Q., Wu X., Zheng J., Pan X., Peng J. (2005). Peroxisome proliferator activated receptor-gamma ligands induced cell growth inhibition and its influence on matrix metalloproteinase activity in human myeloid leukemia cells. Cancer Chemother. Pharmacol..

[27] Hetzel M., Walcher D., Grüb M., Bach H., Hombach V., Marx N. (2003). Inhibition of MMP-9 expression by PPARγ activators in human bronchial epithelial cells. Thorax.

[28] Biehler E., Alkerwi A., Hoffmann L., Krause E., Guillaume M., Lair M.-L., Bohn T. (2012). Contribution of violaxanthin, neoxanthin, phytoene and phytofluene to total carotenoid intake: Assessment in Luxembourg. J. Food Compos. Anal..

[29] Wawrzyniak A., Hamulka J., Friberg E., Wolk A. (2013). Dietary, anthropometric, and lifestyle correlates of serum carotenoids in postmenopausal women. Eur. J. Nutr..

[30] Fraser G.E., Jaceldo-Siegl K., Henning S.M., Fan J., Knutsen S.F., Haddad E.H., Sabaté J., Beeson W.L., Bennett H. (2016). Biomarkers of dietary intake are correlated with corresponding measures from repeated dietary recalls and food-frequency questionnaires in the adventist health study-2. J. Nutr..

[31] Eroglu A., Harrison E.H. (2013). Carotenoid metabolism in mammals, including man: Formation, occurrence, and function of apocarotenoids. J. Lipid Res..

[32] Krinsky N.I., Johnson E.J. (2005). Carotenoid actions and their relation to health and disease. Mol. Aspects Med..

[33] Krinsky N.I., Yeum K.-J. (2003). Carotenoid-radical interactions. Biochem. Biophys. Res. Commun..

[34] Pradeep C.R., Kuttan G. (2003). Effect of β-Carotene on the inhibition of lung metastasis in mice. Phytomedicine.

[35] Kim D., Lim J.W., Kim H. (2019). β-Carotene inhibits expression of c-myc and cyclin E in *Helicobacter pylori*-infected gastric epithelial cells. J. Cancer Prev..

[36] Jang S.H., Lim J.W., Kim H. (2009). Beta-carotene inhibits *Helicobacter pylori*-induced expression of inducible nitric oxide synthase and cyclooxygenase-2 in human gastric epithelial AGS cells. J. Physiol. Pharmacol..

[37] Cui Y., Lu Z., Bai L., Shi Z., Zhao W.-E., Zhao B. (2007). *β*-Carotene induces apoptosis and up-regulates peroxisome proliferator-activated receptor gamma expression and reactive oxygen species production in MCF-7 cancer cells. Eur. J. Cancer.

[38] Ngoc N.B., Lv P., Zhao W.E. (2018). Suppressive effects of lycopene and β-carotene on the viability of the human esophageal squamous carcinoma cell line EC109. Oncol. Lett..

[39] Kushlinskii N.E., Gershtein E.S., Ivannikov A.A., Davydov M.M., Chang V.L., Ognerubov N.A., Stilidi I.S. (2019). Clinical Significance of Matrix Metalloproteinases in Blood Plasma of Patients with Gastric Cancer. Bull. Exp. Biol. Med..

[40] Garrington T.P., Johnson G.L. (1999). Organization and regulation of mitogen-activated protein kinase signaling pathways. Curr. Opin. Cell Biol..

[41] Kolch W. (2000). Meaningful relationships: The regulation of the Ras/Raf/MEK/ERK pathway by protein interactions. Biochem. J..

[42] Karin M. (1995). The regulation of AP-1 activity by mitogen-activated protein kinases. J. Biol. Chem..

[43] Leppa S., Saffrich R., Ansorge W., Bohmann D. (1998). Differential regulation of c-Jun by ERK and JNK during PC12 cell differentiation. EMBO J..

[44] Finkel T., Holbrook N.J. (2000). Oxidants, oxidative stress and the biology of ageing. Nature.

[45] Clerk A., Michael A., Sugden P.H. (1998). Stimulation of multiple mitogen-activated protein kinase sub-families by oxidative stress and phosphorylation of the small heat shock protein, HSP25/27, in neonatal ventricular myocytes. Biochem. J..

[46] Park Y., Choi J.Y., Lim J.W., Kim H. (2015). β-Carotene-induced apoptosis is mediated with loss of Ku proteins in gastric cancer AGS cells. Genes Nutr..

[47] Jang S.H., Lim J.W., Kim H. (2009). Mechanism of β-carotene induced apoptosis of gastric cancer cells: Involvement of Ataxia-Telangiectasia-Mutated. Ann. N. Y. Acad. Sci..

[48] Palozza P., Serini S., Torsello A., Di Nicuolo F., Piccioni E., Ubaldi V., Pioli C., Wolf F.I., Calviello G. (2003). β-carotene regulates NF-κB DNA-binding activity by a redox mechanism in human leukemia and colon adenocarcinoma cells. J. Nutr..

[49] Park Y., Lee H., Lim J.W., Kim H. (2019). Inhibitory Effect of β-carotene on *Helicobacter pylori*-induced TRAF expression and hyper-proliferation in gastric epithelial cells. Antioxidants.

[50] Ribeiro D., Sousa A., Nicola P., Ferreira de Oliveira J.M.P., Rufino A.T., Silva M., Freitas M., Carvalho F., Fernandes E. (2020). β-Carotene and its physiological metabolites: Effects on oxidative status regulation and genotoxicity in in vitro models. Food Chem. Toxicol..

[51] Kim Y.S., Lee H.A., Lim J.Y., Kim Y., Jung C.H., Yoo S.H., Kim Y. (2014). β-Carotene inhibits neuroblastoma cell invasion and metastasis in vitro and in vivo by decreasing level of hypoxia-inducible factor-1α. J. Nutr. Biochem..

[52] Minami Y., Kawabata K., Kubo Y., Arase S., Hirasaka K., Nikawa T., Bando N., Kawai Y., Terao J. (2009). Peroxidized cholesterol-induced matrix metalloproteinase-9 activation and its suppression by dietary β-carotene in photoaging of hairless mouse skin. J. Nutr. Biochem..

[53] Wertz K., Seifert N., Hunziker P.B., Riss G., Wyss A., Lankin C., Goralczyk R. (2004). β-carotene inhibits UVA-induced matrix metalloprotease 1 and 10 expression in keratinocytes by a singlet oxygen-dependent mechanism. Free Radic. Biol. Med..

[54] Guruvayoorappan C., Kuttan G. (2007). β-Carotene inhibits tumor-specific angiogenesis by altering the cytokine profile and inhibits the nuclear translocation of transcription factors in B16F-10 melanoma cells. Integr. Cancer Ther..

[55] Pham D.N.T., Leclerc D., Lévesque N., Deng L., Rozen R. (2013). β, β-Carotene 15,15′-monooxygenase and its substrate β-carotene modulate migration and invasion in colorectal carcinoma cells. Am. J. Clin. Nutr..

[56] Kaliappan G., Nagarajan P., Moorthy R., Selvi S.K.G., Raj T.A., Kumar J.M. (2013). Ang II induce kidney damage by recruiting inflammatory cells and up regulates PPAR gamma and Renin 1 gene: Effect of β-carotene on chronic renal damage. J. Thromb. Thrombolysis.

[57] Mao Q.-Q., Xu X.-Y., Shang A., Gan R.-Y., Wu D.-T., Atanasov A.G., Li H.-B. (2020). Phytochemicals for the prevention and treatment of gastric cancer: Effects and mechanisms. Int. J. Mol. Sci..

